# Auditory Perceptual Learning for Speech Perception Can be Enhanced by Audiovisual Training

**DOI:** 10.3389/fnins.2013.00034

**Published:** 2013-03-18

**Authors:** Lynne E. Bernstein, Edward T. Auer, Silvio P. Eberhardt, Jintao Jiang

**Affiliations:** ^1^Communication Neuroscience Laboratory, Department of Speech and Hearing Science, George Washington UniversityWashington, DC, USA

**Keywords:** audiovisual speech processing, audiovisual speech perception, perceptual learning, reverse hierarchy theory, auditory perception, visual speech perception, multisensory processing, plasticity and learning

## Abstract

Speech perception under audiovisual (AV) conditions is well known to confer benefits to perception such as increased speed and accuracy. Here, we investigated how AV training might benefit or impede auditory perceptual learning of speech degraded by vocoding. In Experiments 1 and 3, participants learned paired associations between vocoded spoken nonsense words and nonsense pictures. In Experiment 1, paired-associates (PA) AV training of one group of participants was compared with audio-only (AO) training of another group. When tested under AO conditions, the AV-trained group was significantly more accurate than the AO-trained group. In addition, pre- and post-training AO forced-choice consonant identification with untrained nonsense words showed that AV-trained participants had learned significantly more than AO participants. The pattern of results pointed to their having learned at the level of the auditory phonetic features of the vocoded stimuli. Experiment 2, a no-training control with testing and re-testing on the AO consonant identification, showed that the controls were as accurate as the AO-trained participants in Experiment 1 but less accurate than the AV-trained participants. In Experiment 3, PA training alternated AV and AO conditions on a list-by-list basis within participants, and training was to criterion (92% correct). PA training with AO stimuli was reliably more effective than training with AV stimuli. We explain these discrepant results in terms of the so-called “reverse hierarchy theory” of perceptual learning and in terms of the diverse multisensory and unisensory processing resources available to speech perception. We propose that early AV speech integration can potentially impede auditory perceptual learning; but visual top-down access to relevant auditory features can promote auditory perceptual learning.

## Introduction

In addition to the classically defined, high-level multisensory cortical association areas such as the superior temporal sulcus (Calvert et al., 2000; Beauchamp et al., 2004; Miller and D’Esposito, 2005; Nath and Beauchamp, 2012), multisensory processing sites have been identified at lower levels, such as primary or secondary cortical areas and the major thalamic relay nuclei (for reviews, see Foxe and Schroeder, 2005; Driver and Noesselt, 2008; Falchier et al., 2012; Kayser et al., 2012). For example, monkey studies have found visual neuronal inputs to primary auditory cortex and to the caudal auditory belt cortex (Schroeder and Foxe, 2002; Ghazanfar et al., 2005; Kayser et al., 2009). Evidence is also available for auditory neuronal inputs to primary visual cortex (Falchier et al., 2001, 2012). Extensive multisensory connectivity has led to the suggestion that all cortical operations are potentially multisensory (Ghazanfar and Schroeder, 2006).

There is no doubt that speech perception makes use of diverse multisensory cortical processing resources (Sams et al., 1991; Calvert et al., 2000; Möttönen et al., 2002; Miller and D’Esposito, 2005; Saint-Amour et al., 2007; Skipper et al., 2007; Bernstein et al., 2008a,b; Nath and Beauchamp, 2011, 2012), and that visual speech stimuli integrate with auditory stimuli under a wide range of listening conditions and for a wide range of functions. For example, when auditory speech stimuli are degraded, being able to see the talker typically leads to improved perceptual accuracy (e.g., Sumby and Pollack, 1954; MacLeod and Summerfield, 1987; Iverson et al., 1998; Ross et al., 2007; Ma et al., 2009). But even when the auditory stimuli are not degraded, visual speech stimuli can affect speech perception and comprehension. Comprehension of difficult verbal materials can be easier under audiovisual (AV) conditions (Reisberg et al., 1987); Perception in a second language can be more accurate with AV stimuli than with auditory-only stimuli (Hazan et al., 2006); and Numerous demonstrations of the McGurk effect (McGurk and MacDonald, 1976) have shown that when auditory and visual speech consonants are mismatched, perceivers often hear a consonant that is different from either the auditory or visual stimulus *per se* (e.g., Green and Kuhl, 1989; Sekiyama and Tohkura, 1991; Jiang and Bernstein, 2011). The study reported here addressed how training with AV speech stimuli might affect auditory perceptual learning of a type of novel degraded acoustic speech stimulus. At issue was how multisensory resources are deployed in the context of unisensory perceptual learning.

**Figure 1:**
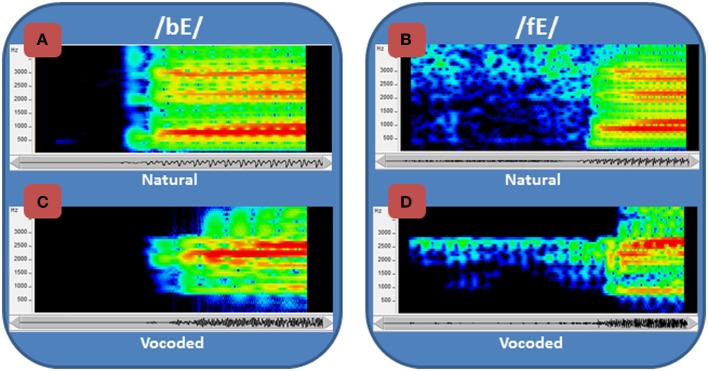
**Spectrograms of normal and vocoded speech**. Spectrograms of speech show the concentrations of energy in the spectra over time. Two speech tokens, /bE/ and /fE/ (i.e., the vowel in “bet”), are shown in spectrograms of the natural **(A)** and **(B)** recorded speech and the vocoded **(C)** and **(D)** speech. The frequency range of the spectrograms is restricted to 4 kHz, because all of the energy from the vocoder is similarly limited. The amplitudes are represented as a heat map, with red the highest amplitude and dark blue the lowest. In addition to representing the speech as the sum of sinewaves at the center of each vocoder filter (see text), the vocoder also tilted the spectrum so that it did not roll off at approximately 6 dB/octave, which is natural to speech. Thus, the amplitudes of the frequencies vary across the natural and the vocoded speech, in addition to the frequency ranges and spectral detail.

This study focused on learning to perceive degraded acoustic speech. The spoken nonsense words that were used as stimuli were transformed by passing them through a vocoder, a signal-processor that systematically degrades the speech (Iverson et al., 1998; Scott et al., 2000) and typically requires experience or training to achieve improved levels of perceptual accuracy (e.g., Davis et al., 2005; Scott et al., 2006; Hervais-Adelman et al., 2011). The vocoder here transformed fine-grained acoustic spectral cues, including vocal tract resonance changes that are cues to phoneme (consonants and vowels) distinctions, into coarse spectral cues by coding energy in 15 frequency bands as amplitudes of fixed-frequency sinusoids at the center frequency of each band (Figure 1). In addition, the normal speech spectrum, which falls off at approximately 6 dB per octave, was tilted so that amplitudes in vocoder bands were approximately equalized. Figure 1 shows spectrograms of the syllables /bE/ and /fE/ (i.e., the vowel in “bet”) for the natural recorded speech (Figures 1A,C) and the vocoded speech (Figures 1B,D). The vocoding highly reduces the available acoustic information, emphasizes the second speech formant (vocal tract resonance), known to be highly informative for speech perception (Liberman et al., 1967), and reduces or omits the first and third formants, which are also important.

We hypothesized that information in visual speech stimuli can provide top-down guidance for auditory perceptual learning (Ahissar and Hochstein, 1997; Kral and Eggermont, 2007; Ahissar et al., 2008) of the cues to phoneme perception in the vocoded acoustic signals. That is, in addition to integrating with auditory speech cues during perception, visual speech stimuli were hypothesized to be able to guide auditory perceptual learning, with the result that auditory-only perception is improved more following AV than following auditory-only training. Our rationale for this hypothesis about the benefits of visual speech is that certain visual speech features can be reliably available (Bernstein et al., 2000; Bernstein, 2012), and they are correlated in real time with auditory features (Yehia et al., 1998; Jiang et al., 2002; Jiang and Bernstein, 2011). Therefore, they could help to train novel or unfamiliar vocoded auditory speech features when they are available during training. For example, /f/ and /b/ are visually distinctive (Auer and Bernstein, 1997), but the distinction between vocoded /f/ and /b/, which is available in the novel acoustic signals (see Figures 1B,D), might not be discerned without training. Training with the AV stimuli could enhance auditory perceptual learning, because the visual features that are integrated during visual perceptual processing (Bernstein et al., 2011; Bernstein, 2012) could be used to guide top-down attention to the correlated auditory cues that discriminate /f/ from /b/. In contrast, training with auditory-only stimuli contributes no additional information for learning novel cues or features, beyond what can be gleaned from merely repeating the stimulus, and the perceiver might not learn to distinguish the critical novel cues. Alternatively, early integration of auditory and visual speech features could impede auditory perceptual learning, because perception would be successful without accessing the available auditory distinctions in the vocoded stimuli.

In the study reported here, we compared auditory perceptual learning based on training with AV versus audio-only (AO) speech stimuli. Because our hypothesis concerned perceptual learning of acoustic speech features, the experimental task had to preclude access to pre-existing lexical knowledge, a type of high-level representation, that could function like visual speech stimuli. Lexical knowledge itself can be a top-down source for auditory perceptual learning (Davis et al., 2005). Therefore, all of the stimuli in the study were spoken nonsense words. Auditory training was given in a paired-associates (PA) task. Participants learned paired associations between disyllabic spoken nonsense words and nonsense pictures. Training was under AV and/or AO conditions, and testing was exclusively under AO conditions. In addition to PA training and testing, a forced-choice identification paradigm was used to test auditory consonant identification before and after training, using stimuli that were not used in training. The consonant identification also served to test for generalization to new stimuli in a different perceptual task and to infer the level of auditory perceptual learning that was achieved. Our results show that AV training can significantly benefit auditory perceptual learning beyond AO training. But the details of the training protocol appear to be critically important to achieving benefit from visual stimuli, because AV training can also lead to poorer AO performance. In our General Discussion, we propose a model of how AV stimuli can guide auditory perceptual learning through top-down visual access to useful auditory distinctions; or how AV stimuli can impede auditory perceptual learning through early immediate integration of auditory and visual speech cues.

## Materials and Methods

### Experiment 1 between-participant training with fixed numbers of training trials

In Experiment 1, participants were assigned to either AV or AO PA training followed by AO testing. Training in the PA task used nonsense pictures and nonsense words of the form consonant-vowel-consonant-vowel-consonant (CVCVC), modeled on the phonotactics of disyllabic English words. The PA task emulated the learning of new vocabulary items. Thus, participants were required to learn at multiple levels, including the perceptual (novel acoustic transform and novel lexical word form) and the high-level associative (semantic association between word form and picture). Here, participants were tested on the number of paired associations they could demonstrate following training. If AV-trained participants were more successful during AO testing than AO-trained participants, who had achieved equivalent performance during training, then the implication would be that the AV-trained participants learned more about the auditory stimuli. Pre- and post-training forced-choice consonant identification was tested, using an untrained set of CVCVC nonsense words. The identification measures were the number of correctly identified consonants in the three positions of the nonsense words. If differential learning occurred across the position of the consonant in the word, then the implication would be that participants learned sub-phonemic auditory features, because acoustic phonetic signals differ across segment position in a word (Stevens, 1998).

#### Subjects

Individuals were screened for American English as a first language, normal or corrected-to-normal vision in each eye of 20/30 or better (using a Snellen chart), and hearing (25 dB HL or better in each ear for frequencies between 125 and 8 KHz, using an Audiometrics GSI 16 audiometer with insert earphones). The experiment was carried out at two different locations, using the same equipment and procedures. At the House Research Institute (Los Angeles, CA, USA), 12 volunteers, ages 18–48 years (mean = 30 years), including six males, completed the experiment, and an additional five volunteers were asked to discontinue the experiment after they were mistakenly presented with non-distorted speech. At the George Washington University, 25 volunteers, ages 19–30 (mean = 22), including five males, completed the experiment, and an additional four dropped out due to lack of availability. In all, 18 participants completed AV training, and 19 completed AO training. They were paid $12 per hour of testing, plus any travel expenses incurred. Subjects gave written consent. Human subject participation was approved by either the St. Vincent’s Hospital Institutional Review Board (Los Angeles, CA, USA) or by the George Washington University Institutional Review Board (Washington, DC, USA).

#### Stimuli

##### Speech

The spoken CVCVC nonsense words were modeled on English phonotactics (i.e., the sequential speech patterns in English). They were visually distinct for lipreading and visually unique from real English words (i.e., the words were designed to not be mistaken as real words, if they were lipread without accompanying audio). Thus, for example, the nonsense word *mucker* was not included in the set, because the visual stimulus could be mistaken for the real word *pucker*, inasmuch as the phonemes /p, m/ are visually highly similar (Auer and Bernstein, 1997).

The process of stimulus generation was as follows. Syllables with the structure CV-, -VCV-, and –VC were extracted from the 35,000-word phonemically transcribed PhLex database (Seitz et al., 1998). Based on empirically derived phonotactic probabilities, a Monte Carlo simulation was used to generate 30,000 CVCVC candidate nonsense words, which were then further processed. First, existing visual phoneme confusion data were used to model the confusability of the phonemes (Auer and Bernstein, 1997; Iverson et al., 1998). Then the candidate nonsense words were computationally processed, taking into account their visual confusability with real words and other nonsense words (Auer and Bernstein, 1997). Stimuli that would have been easily confused by vision were grouped into sets, and only one CVCVC word was chosen from each set, with the requirements that (1) the final set of nonsense words would include all the English phonemes, and (2) within each CVCVC, the five phonemes would be visually distinct to a lipreader (Auer and Bernstein, 1997). These constraints implied that within a list of nonsense words, visual information should be sufficient to differentiate among items.

The female talker whose data were used to model consonant and vowel confusability was the same talker used to produce the nonsense words. She was professionally videotaped uttering the final set of 260 CVCVC words.

Stimulus lists were constructed by first ordering stimuli by initial consonant and vowel, and then dividing the list on even- versus odd-numbered items to form two lists from which items were randomly selected. Two 49-item lists were selected for the pre- and post-training consonant identification task (Table 1; see Table 2 for transcription key). Two six-item lists were selected from 12-item lists for pre- and post-training practice. Six lists of 12 items for PA training and six lists of six items as new items during PA testing were selected from the remaining available words (Table 3).

**Table 1:** **Pre-test and post-test consonant identification lists in single-phoneme transcription format**.

List 1		List 2	
banoz	pETat	batok	pod∧n
biscg	ponRs	Bizxd	pUrIn
brcit	pUtIl	bRsxv	Ribcg
bulad	rid∧t	bUnxl	rob∧l
c@GRz	rot∧k	C@pRk	s@naJ
ccrik	s@vxk	CctIG	SIGRt
cEmxl	sik∧S	CEvxs	SInal
deman	Sivab	Dumxs	sRbik
duzxn	sRmaS	fRCxl	Sulak
fRsal	suZxm	gInxz	t@Cig
gIZxn	t@n∧m	h@n∧p	tEmaS
h@nus	tErin	Jcrat	Tib∧n
jcrib	Tis∧p	JEnap	Tufxl
jEris	Tukad	JozIG	v@sap
junxs	vEJUd	k@Cud	vEJxn
k@Taz	vob∧n	Kcrit	vomit
kctas	vRbIG	m@DRz	vRlIs
m@JUd	Wcfxn	madRz	wct∧m
makiz	wEJxk	Mckit	wEkab
mczin	wRk∧l	mEros	wRlas
mezxl	Yizxk	nECUt	yiZxs
Net∧m	yUbIg	Nobad	yUmEs
noluz	Yusap	p@Cik	yutIb
p@Tan	zobIG	paJUt	zoSxn
palIt		pEluz	

*Words are transcribed, because English orthography does not map uniquely to English phonemes. Table 2 gives the phoneme transcription key. Lists 1 and 2 were randomly selected on a per-subject basis for use in pre-test and post-test (or test, re-test) consonant identification tasks. The practice list (JUkiz, zIJxl, dISus, JEroz, mivRd, DEkxs) was used before each test to ensure that participants understood the task*.

**Table 2:** **Transcription keys for nonsense word consonants and vowels**.

Consonant sounds represented by lower case on keyboard		Consonant sounds represented by UPPER case on keyboard	
**A**			
**Consonant**	**Example**	**Consonant**	**Example**
b	(b)ut	C	su(ch)
d	goo(d)	D	(th)at
f	(f)ew	G	lo(ng)
g	(g)ood	J	lar(g)e
h	(h)is	S	(sh)e
k	(c)an	T	bo(th)
l	(l)ike	Z	u(s)ual
m	(m)ore		
n	(n)ew	consonants easily confused	
p	(p)ut	D	T
r	(r)oom	s	S
s	(s)ome	g	G
t	bu(t)	z	Z
v	gi(v)e	c	J
w	(w)ill	k	
y	(y)ou		
z	wa(s)		
**B**			
**Vowel**	**Example**	**Vowel**	**Example**
a	b(o)b	@	b(a)t
o	b(oa)t	E	b(e)t
i	b(ea)t	x	(a)bout
c	b(ou)ght	u	l(u)te
r	b(ir)d	I	b(i)t
u	b(oo)k	∧	b(u)t

*(A) Consonant transcription key. (B) Vowel transcription key. These transcription keys were used to assign a single orthographic symbol for each English consonant and vowel phoneme in the nonsense words listed in Tables 1 and 3. The consonant transcription key was used to train and test participants to carry out forced-choice consonant identification*.

**Table 3:** **Word lists for paired-associates task. Lists 1–4 were used in Experiment 1**.

Training list 1	Test list 1	Training list 2	Test list 2	Training list 3	Test list 3	Training list 4	Test list 4
sICUd	sICUd	mITak	mITak	hIluz	hIluz	kizxl	Kizxl
pcriD	pcriD	lRman	lRman	Cudxk	Cudxk	wEsIk	wEsIk
CRfIG	CRfIG	Sczxn	Sczxn	bUran	bUran	Bincl	Bincl
wInct	wInct	Bodut	Bodut	Jobxt	Jobxt	Pcgxs	Pcgxs
kUmxl	kUmxl	Ridap	Ridap	m@fis	m@fis	TuSxz	TuSxz
hUbIG	hUbIG	zEriC	zEriC	kcraC	kcraC	s@bad	s@bad
digaz	SEsxl	pIDRz	pEt∧f	tEfRk	zEnop	Yupan	m@d∧v
lIZxs	bozEn	wRsIG	f@Jxs	Ncrim	dik∧p	hob∧k	SRfxn
mcTxs	JovRs	k@fRt	viw∧s	ril∧n	yUS∧k	dISxp	l@kat
tETan	m@tuT	TEmat	nIsxJ	TIfxs	rIZxl	vIpxd	zESxm
rip∧J	fctab	dib∧J	JUkiz	fICUt	Lctak	m@Jxv	CIlxz
Yulat	D@zxk	sEJud	wEsxJ	S@dxz	w@vxt	Nupis	fEkRz

**Training list 5**	**Test list 5**	**Training list 6**	**Test list 6**			**Practice list 1**	**Practice list 2**

zudxn	Zudxn	mEzud	mEzud			fISxb	hRsak
wizcg	Wizcg	bikud	bikud			ballot	pEJun
m@nad	m@nad	SIzxv	SIzxv			yUtin	bUris
C@zxd	C@zxd	hivan	hivan			mRsaC	JEroz
pincg	Pincg	vid∧n	vid∧n			DEkxs	pEvxk
y@pat	y@pat	JIfxl	JIfxl			bon∧f	Mizcl
b@GIt	k@tup	nimat	pEriT			zErIp	dISus
hozIk	gIsan	pasIk	naSis			ripEs	dipcs
lipRt	h@Jus	rigab	kRCxm			hISxd	vRpad
fcris	Sigak	tcrab	gEsak			hon∧t	mivRd
nopiz	Fonab	k@pIG	wimun			hImut	dIs∧f
rik∧f	rEmRz	wilus	zIJxl			p@fxJ	wEvRz

Practice List 1 was used to familiarize participants with the task. Lists 1–3 were used for AO training and testing, and Lists 4–6 for AV training and AO testing. Practice List 2 was presented AO, and Practice List 1 was presented AV. Test lists always show that the first six words in the list were carried into testing and six new words were substituted for six trained words. (Table 2 gives the transcription key for phoneme mappings.)

The acoustic speech stimuli were processed through a custom realtime hardware/software vocoder (Iverson et al., 1998). The vocoder detected speech energy in thirteen 120-Hz-bandwidth bandpass filters with center frequencies every 150 Hz from 825 Hz through 2625 Hz. Two additional filters were used to convey high frequencies. One was a bandpass filter centered at 3115 Hz with 350 Hz bandwidth and the other a highpass filter with 3565 Hz cutoff. The energy detected in each band was used to amplitude-modulate a fixed-frequency sinewave at the center frequency of that band (and at 3565 Hz in the case of the highpass filter). The sum of the 15 sinewaves comprised the vocoded acoustic signal. This acoustic transformation retained the gross spectral-temporal amplitude information in the waveform while eliminating finer distinctions such as fundamental frequency variations and eliminating the natural spectral tilt of the vocal tract resonances. Figure 1 compares /ba/ and /fa/ between the original recordings and the vocoded versions.

##### Nonsense pictures

Nonsense pictures in the PA task were from the “fribble” image set (Databases/TarrLab/(http://wiki.cnbc.cmu.edu/Novel_Objects)). Fribbles comprise 12 species with distinct body “core” shape and color, with 81 exemplars per specie obtained by varying the forms of each of four appendage parts. From the available images, 13 lists of 12 images each were created such that each list used three different body forms and no duplicated appendage forms, rendering the images within each list highly distinctive (Williams and Simons, 2000). No appendage was repeated across lists.

#### Design

**Figure 2:**
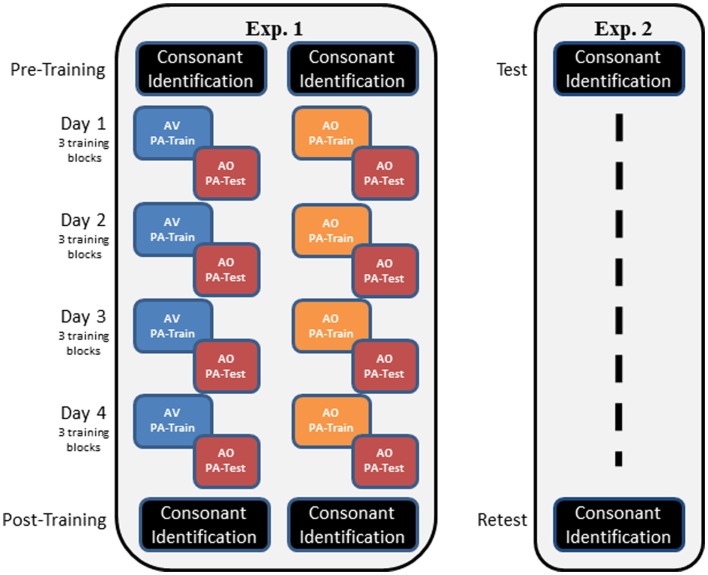
**Overall designs of Experiments 1 and 2**. In Experiment 1, participants carried out pre-training consonant identification, followed by either AV or AO training on four stimulus lists, with AO tests on each list. Training of three blocks per list was carried out on a separate day for each list. Post-training, participants were tested again on AO forced-choice consonant identification. In Experiment 2, participants were tested only on forced-choice consonant identification on two different days (test, re-test). The two administrations of the forced-choice consonant identification used different stimulus lists.

Figure 2 outlines the overall design of the experiment. Participants completed pre-training consonant identification familiarization and pre-training forced-choice consonant identification. Then, on each of four different days, they completed three blocks of PA training and AO testing associated with one word list. Participants were assigned to either AV or AO training for the duration of the experiment. Following the PA training and testing, participants were tested again on AO forced-choice consonant identification.

##### Consonant identification familiarization procedure

The pre- and post-training forced-choice consonant identification involved all the English consonants. Because English orthography is not uniquely mapped to English phonemes, participants were first familiarized with the orthographic transcription system, which was compatible with single-character keyboard entry. An answer key (the consonants listed in Table 2), also available during testing, was used to explain the orthographic system. During familiarization, participants filled out two self-scored worksheets, one with the key available and one without. The participants’ task was to transcribe 48 consonants in real English words while looking at the key and then 71 consonants in real words without looking at the key. A six-item practice test was randomly selected from two practice lists. All the participants were able to use the orthographic transcription system.

##### Pre- and post-training test procedure

Audio-only forced-choice consonant identification was carried out with CVCVC nonsense words. On each trial, following presentation of a stimulus, a response string of the form “__-__-__” appeared on the monitor, and the participants typed, in order, the three consonants that they had perceived in the AO spoken stimulus. They were instructed to guess when necessary. Only characters from the response set were displayed in the response string. It was possible to correct a response, and use of the enter key completed the trial. No feedback was given for the correctness of the responses. Different test lists were assigned across pre- and post-training testing, and list order was counter-balanced across participants.

##### Paired-associates training procedure

**Figure 3:**
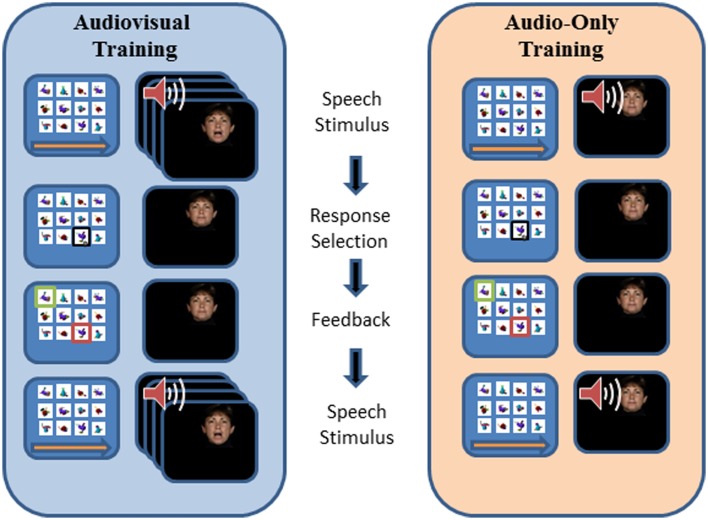
**Trial structure for paired-associates training**. A speech stimulus was presented, followed by the participant’s response selection, followed by feedback and a repetition of the speech stimulus. Each panel depicts the screen showing the fribble images side-by-side with the video monitor showing the talker. The trial structure for AV and AO training followed the same sequence, except that during AV training the video was played synchronously with the audio, and during AO training a still neutral face was played during the audio.

Figure 3 outlines the design of a PA training trial. During training, the participant’s task was to learn, with feedback over repeated presentations, lists of individual associations between 12 fribble images and 12 CVCVC vocoded spoken nonsense words. In Figure 3, an AV training trial is shown in the left column and an AO training trial is shown in the right column. Each trial began with a computer-monitor display of the 12-fribble image matrix (three rows of four columns, with image position within the matrix randomly selected on a trial-by-trial basis). During AV training, a video of the talker was played in synchrony with the spoken audio, and during AO training, a single still image of the talker’s face was displayed on the monitor during audio presentation. The talker was presented on a different monitor than the fribble matrix monitor, and a large arrow appeared on the bottom of the fribble monitor pointing left to remind the participant to focus attention on the talker. The participant used the computer mouse to choose a fribble image following the speech stimulus. Feedback was given by outlining the correct fribble in green and an incorrect choice in red. After a short interval, the speech stimulus was always repeated, while the fribble images and borders remained unchanged. A training block comprised two repetitions of the 12 paired associations in pseudorandom order. Prior to the first training list in each condition (AV or AO), participants were given practice with one block of six trials.

##### Paired-associate testing procedure

paired-associates testing immediately followed training. The testing procedure was the same as that of PA training, except the stimuli were always AO, no feedback was given, the stimulus was not repeated during the trial, and each response triggered the next trial. Six of the trained spoken words and all 12 of the fribble images were used for testing. The associations for the six retained words were unchanged. Six new nonsense words were paired with the fribble images of the discarded words. A testing block comprised, in pseudorandom order, one presentation of the 12 stimuli, and three blocks were presented. The test score was the proportion of correct paired associations of trained words.

##### Apparatus

Audiovisual CVCVC tokens were digitized, edited, and conveyed to digital video disk (DVD) format. The acoustic waveforms were vocoded in real time, and the audio stimuli were output at a calibrated 65 dB A-weighted sound pressure level (SPL) using a JBL LSR6325P-1 loudspeaker. Participants were tested in an Industrial Acoustics Company (IAC) double-walled sound-attenuating booth using a standard computer interface that included a 51 cm LCD monitor, and a 35.6 cm Sony PVM-14N5U NTSC video monitor for display of speech video from the DVD. Monitors were located about 1 m from the participant’s eyes, so that the computer-monitor subtended a visual angle of 23.1° horizontally and 17.3 vertically with the 12 fribble matrix filling the monitor. The visual speech was displayed on the NTSC monitor with the talker’s head subtending visual angles of 3.9° horizontally and 5.7 vertically. Custom software was used to run the experiment.

##### Analyses

In order to stabilize the variance of proportion correct scores, the arcsin transformation, $X^{1}=\sin^{-1}\sqrt{X}$ was computed, where *X* was the proportion correct score computed over the appropriate set of trials. All analyses were also conducted in parallel on untransformed scores, and all of the parallel analyses agreed. Statistics are reported on the arcsin transformed data, but tables, means, and figures are untransformed to facilitate interpretation.

#### Results and discussion

##### Paired-associates training

Initial inspection of the training and testing data showed there to be wide individual variation. There were participants who were unable to learn associations to an acceptably high-level of accuracy within the three training blocks. In order to assure that a relatively similar level of PA learning had taken place across training conditions, the criterion of at least 75% correct on the third training block was set for use of a participant’s data. That is, we chose to remove the data sets obtained from participants who appeared to have difficulty learning associations *per se*. This criterion removed data from 10 participants from analyses. An additional participant was dropped because of scoring 6% correct on the test of one list, deviating greatly from typical test performance (mean = 94%, minimum = 67%, maximum = 100%). The analyses reported henceforth are on the data from 25 participants, 12 in the AV-trained group and 13 in the AO-trained group.

To examine performance during training, scores were submitted to RMANOVA with the within subjects factors of training list (1–4) and training block (1–3), and the between-subjects factor of training group (AO-trained, AV-trained). Importantly, no evidence was obtained for a reliable main effect or interaction with training group. Reliable main effects were obtained for training list *F*(3, 69) = 19.26, MSE = 0.49, *p* < 0.001, $\eta_{p}^{\text{2}}=0.46$, and training block, *F*(2, 46) = 651.09, MSE = 14.41, *p* < 0.001, $\eta_{p}^{\text{2}}=0.97$. A significant interaction between list and block (see Table 4), *F*(6, 138) = 6.77, MSE = 0.08, *p* < 0.001, $\eta_{p}^{\text{2}}=0.23$, was also obtained. Table 4 shows that, with experience, learning was faster.

**Table 4:** **Experiment 1 training scores as a function of list and block**.

	Block 1	Block 2	Block 3
List 1	31(2.0)	76(3.3)	95(1.3)
List 2	42(2.2)	90(2.0)	98(0.8)
List 3	49(2.5)	93(1.6)	96(1.2)
List 4	51(2.1)	91(1.8)	97(1.0)

*The means are presented with the standard error of the mean in parenthesis*.

#### Paired-associates test results

The critical question was whether the AV-trained participants were more accurate than AO-trained participants when both were tested with AO stimuli. The proportion correct PA test scores based on three repetitions of each of the six trained items was computed. The values were submitted to RMANOVA with the within subject factor of training list (1–4) and the between subject factor training condition (AO, AV). A main effect of training condition, *F*(1, 23) = 7.619, MSE = 0.36, *p* < 0.05, $\eta_{p}^{\text{2}}=0.25$, was obtained. The AV-trained participants had higher AO test scores (97% correct test scores, SE = 1.4) than did the AO-trained participants (92% correct test scores, SE = 1.4). No other effects were reliable. The responses to the six untrained words that were presented during testing were also checked for accuracy, and the scores were very low.

#### Pre- and post-training results

Forced-choice consonant identification data were collected pre- and post-training on independent lists of AO nonsense words. Proportion correct identification scores for consonants in initial, medial, and final position were computed separately on pre- and post-training data. Scores were submitted to RMANOVA with within-subject factors of time of testing (pre- versus post-training), consonant position (initial, medial, and final), and between-subjects factor group (AV-trained, AO-trained). The main effects of time of testing, *F*(1, 23) = 141.08, MSE = 0.98, *p* < 0.001, $\eta_{p}^{\text{2}}=0.86$, and of consonant position, *F*(2, 46) = 49.22, MSE = 0.28, *p* < 0.001, $\eta_{p}^{\text{2}}=0.68$, were both reliable.

**Figure 4:**
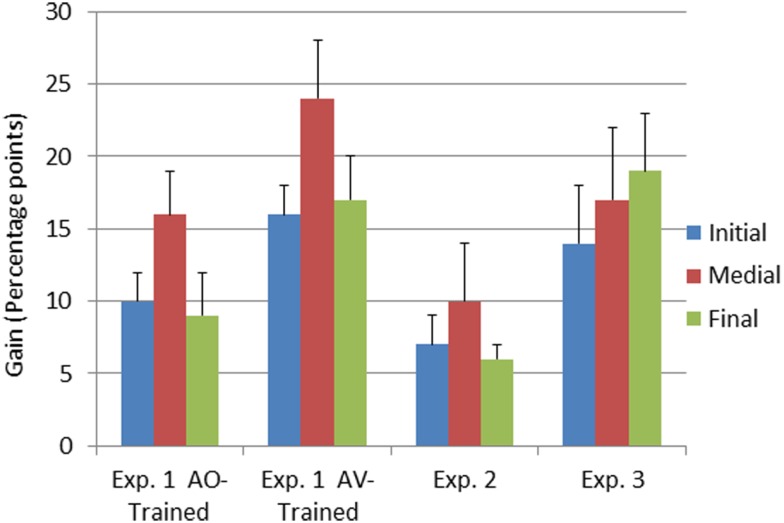
**Pre-to-post-training gain scores as a function of experiment and consonant position**. Gain scores represent the means of the arithmetic difference between first and second forced-choice consonant identification test scores obtained in Experiments 1–3. The error bars represent 1 SE of the mean. Results are shown separately for the three consonant positions in the CVCVC stimuli.

The interaction between time of testing and group was reliable, *F*(1, 23) = 8.54, MSE = 0.06, *p* < 0.05, $\eta_{p}^{\text{2}}=0.27$. The AV-trained participants had lower pre-training forced-choice consonant identification scores and higher post-training scores (AV-trained pre 32% correct, post 50% correct; AO-trained pre 35% correct, post 47% correct), improving on average by 18% points. The AO-trained participants group improved their scores on average by 12% points. Because the two groups were different at pre-training, as well as post-training, post-training − pre-training gain scores were computed and submitted to an independent samples *t-test*. The gains obtained by the AV-trained group were significantly larger than the gains of the AO-trained group, *t*(23) = 2.91, *p* < 0.05 (see Figure 4).

The interaction between time of testing and consonant position was reliable, *F*(2, 46) = 4.49, MSE = 0.02, *p* < 0.05, $\eta_{p}^{\text{2}}=0.16$ (see Table 5). *Post hoc* tests with RMANOVA using the results for the individual consonant positions (initial, medial, and final) revealed that the magnitude of the difference in accuracy between initial and medial consonants was larger post-training than pre-training, *F*(1, 24) = 7.45, MSE = 0.07, *p* < 0.05, $\eta_{p}^{\text{2}}=0.24$, as was the difference between final and medial consonants, *F*(1, 24) = 5.67, MSE = 0.07, *p* < 0.05, $\eta_{p}^{\text{2}}=0.19$. That is, the biggest perceptual learning gains were obtained for medial consonants (see Figure 4). AV-trained participants gained 24% points accuracy for medial consonants, and AO-trained participants gained 17% points.

**Table 5:** **Pre-training and post-training forced-choice consonant identification scores across experiments as a function of consonant position**.

		Consonant Position		
		Initial	Medial	Final
Experiment 1
AO training	Pre-	30 (1.7)	41 (3.7)	34 (2.5)
	Post-	40 (2.2)	58 (3.2)	43 (3.0)
AV training	Pre-	27 (1.7)	37 (3.9)	30 (2.6)
	Post-	43 (2.3)	61 (3.3)	47 (3.1)
Experiment 2	Test	31 (3.2)	44 (3.4)	34 (2.5)
	Re-test	37 (2.5)	54 (4.7)	40 (3.1)
Experiment 3	Pre-	31 (2.4)	47 (4.4)	34 (2.6)
	Post-	46 (4.4)	64 (4.5)	53 (4.1)

*The tabled values are the percent correct means and standard error of the means in parentheses for each of the consonant positions in the CVCVC stimuli. In Experiments 1 and 3, the scores were obtained pre- and post-training. In Experiment 2, the scores were obtained without intervening training (test, re-test)*.

### Experiment 2 no-training control

In Experiment 1, AV training resulted in better AO paired association learning and more accurate forced-choice consonant identification than did AO training. However, the design could not be used to conclude that all gains on the forced-choice consonant identification task were due to training. Therefore, a control experiment was conducted in which the forced-choice consonant identification task was administered twice but *without* intervening training.

#### Materials and methods

##### Subjects

Ten volunteers, aged 22–48 years of age, two male, participated in the experiment. The criteria for inclusion were the same as in Experiment 1.

##### Procedure

Only the brief AO consonant familiarization procedure, practice, pre-training (test), and post-training (re-test) consonant identification tests were administered (Figure 2). The time between test and re-test ranged from 3 to 16 days (mean = 8.1 days). The procedures for administering the forced-choice consonant identification were the same as in Experiment 1.

##### Results and discussion

The test and re-test forced-choice consonant identification data were submitted to RMANOVA with within-subject factors of time of testing (test, re-test) and consonant position (initial, medial, final). The main effects of time of testing, *F*(1, 9) = 24.49, MSE = 0.10, *p* < 0.05, $\eta_{p}^{\text{2}}=0.73$, and of consonant position, *F*(2, 18) = 32.55, MSE = 0.13, *p* < 0.001, $\eta_{p}^{\text{2}}=0.78$, were reliable. There were no reliable interactions. Identification accuracy increased from test (36% correct, SE = 2.7) to re-test (44% correct, SE = 3.1). Linear contrasts revealed that accuracy differed among all three consonant positions (initial = 34%, SE = 2.7; medial = 49%, SE = 3.6; final = 37% correct, SE = 2.7) (see Table 5).

Consonant identification gain scores from Experiments 1 and 2 (Figure 4) were submitted to RMANOVA with the between subject factor training group (AO-trained and AV-trained from Experiment 1 and no-training control from Experiment 2) and the within subject factor consonant position (initial, medial, final). Training group was a reliable factor, *F*(2, 32) = 10.42, MSE = 0.13, *p* < 0.001, $\eta_{p}^{\text{2}}=0.83$. Pair-wise comparisons between AO-trained (Experiment 1), AV-trained (Experiment1), and the no-training control (Experiment 2) showed that AV-trained participants had significantly higher forced-choice consonant identification gain scores than controls (see Figure 4) (*p* < 0.05). But gain scores of Experiment 1 AO-trained participants were not reliably different from those of the no-training controls. Thus, across experiments, only the AV-trained participants demonstrated auditory perceptual learning that was more successful than merely participating in a test-re-test consonant forced-choice identification task.

Consonant position was reliable in the comparison across groups, *F*(2, 64) = 4.37, MSE = 0.04, *p* < 0.05, $\eta_{p}^{\text{2}}=0.12$. Pair-wise comparisons revealed that medial pre-to-post gain scores differed from initial and final gain scores (initial = 11.6%, SE = 1.3; medial = 17.6%, SE = 2.3; final = 11.2%, SE = 2.7; *p* < 0.05).

### Experiment 3 within-participant audiovisual and auditory-only training

**Figure 5:**
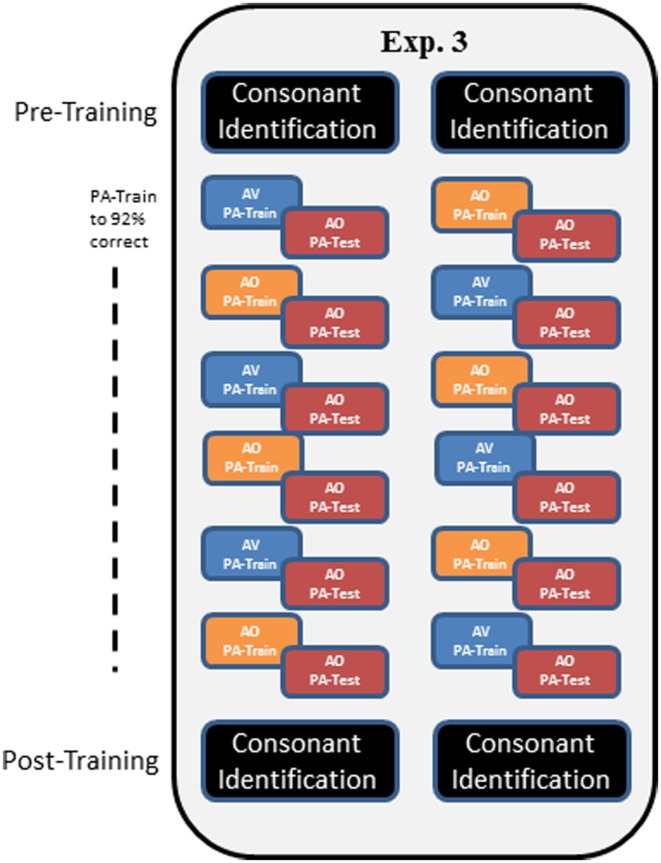
**Overall design of Experiment 3**. In Experiment 3, participants carried out pre-training consonant identification, followed by alternating AV and AO training, counter-balanced across participants as shown by the two columns of train versus test in the figure. Training blocks on each list were repeated until the participant achieved 92% correct. Then AO testing was administered. Post-training, participants were tested again on AO forced-choice consonant identification.

In Experiment 3, a modified training protocol was carried out in order to test whether the AV training advantage in Experiment 1 would be reliable under a different training protocol. Training followed that of Experiment 1, except that participants were trained until they reached the criterion of 92% correct within a training block and list. Also, AV and AO training conditions were alternated across lists, and six lists were trained (Figure 5).

#### Materials and methods

##### Subjects

Fifteen participants were recruited and started the experiment. The criteria for inclusion in the experiment were the same as in Experiment 1. Two dropped out due to difficulty learning the paired associations. The 13 who completed testing were ages 21–51 years (mean = 28 years), with two males.

##### Procedures

Mixed PA AV and AO training was given with counter-balanced initial condition and six lists total (AO, AV, AO, AV, AO, AV, or AV, AO, AV, AO, AV, AO) (see Figure 5). Testing was always AO. Every list of paired associations was trained until the participant scored at least 92% correct. Then, in the same session, the corresponding AO test was administered. Participants were permitted to train on more than one list per session. The forced-choice consonant identification test was administered pre- and post-training as in Experiment 1.

#### Results

##### Paired-associates training

The number of training trials to achieve the 92% correct criterion was submitted to RMANOVA with the within subjects factors of training condition (AO, AV) and list (first, second, third). The main effect of list, *F*(2, 24) = 4.85, MSE = 1602.46, *p* < 0.05, $\eta_{p}^{\text{2}}=0.29$, was the only factor that reached significance. Pair-wise comparisons indicated that, across training condition, more trials (mean = 76.6, SE 6.16) were needed to reach criterion on the first list than on the second (mean = 64.6, SE 5.18) and third (mean = 61.8, SE 5.74) (*p* < 0.05), and the latter two did not differ.

The mean accuracy scores over the blocks to criterion within a list were also submitted to RMANOVA with the within subjects factors of training condition (AO, AV) and list (first, second, third). Again, the main effect of list, *F* (2, 24) = 14.15, MSE = 0.04, *p* < 0.001, $\eta_{p}^{\text{2}}=0.54$, was the only significant factor. Pair-wise comparisons indicated that the first list was less accurate (mean = 66.5, SE = 1.5) than the second (mean = 71.6, SE = 1.8), which was less accurate than the third (mean = 73.9, SE = 1.2; *p* < 0.05).

##### Paired-associates test results

The PA test results were submitted to RMANOVA with within subject factors of training condition (AO, AV) and list (first, second, third). The main effect of training condition was the only significant effect, *F* (1, 12) = 8.44, MSE = 0.25, p < 0.05, $\eta_{p}^{\text{2}}=0.41$. AO-trained PA test scores were higher (94.0% correct mean test score, SE = 1.8) than AV-trained PA test scores (88.9% correct mean test score, SE = 2.5).

In Experiment 1, AV PA training resulted in higher AO test scores (97% correct test scores, SE = 1.4) than did AO training (92% correct AO test scores, SE = 1.4). To compare PA test scores across Experiments 1 and 3 (which had different designs), we pooled test scores within subject separately for AV- and AO-trained lists in each experiment. The results showed that AV training in Experiment 1 was significantly more effective than in Experiment 3, *t* (23) = 2.78, *p* < 0.05. But the AO scores were not different across experiments.

The discrepancy in PA results across Experiments 1 and 3 might have been related to the different criteria for learning that was used to accept data. In Experiment 1, a performance criterion of 75% correct on the third training block for each list was used for inclusion of data. This resulted in dropping 10 out of 36 participants (another one was dropped for an exceptionally low AO test score on trained stimuli). In Experiment 3, two participants were unable to learn the PA stimuli to criterion of 92% correct. However, if we had imposed the 75% correct criterion on the third training block in Experiment 3, 4 out of 13 participants would have failed, which is a comparable proportion to that of Experiment 1. Thus, the results across experiments seem unlikely to be related to group differences in ability to learn paired associations.

##### Pre- and post-training consonant identification

Forced-choice consonant identification scores were submitted to RMANOVA with the within subjects factors of time of testing (pre- versus post-training) and consonant position (initial, medial, final). The main effects of time of testing, *F*(1, 12) = 15.83, MSE = 0.68, *p* < 0.05, $\eta_{p}^{\text{2}}=0.57$, and of consonant position, *F*(2, 24) = 38.99, MSE = 0.23, *p* < 0.001, $\eta_{p}^{\text{2}}=0.77$, were reliable (see Figure 4 and Table 5). The interaction between time of testing and consonant position was not reliable. Consonant identification accuracy increased from pre- (37% correct, SE = 2.7) to post-training (54% correct, SE = 4.1). Linear contrasts revealed that accuracy differed between all three positions (initial = 38%, SE = 2.7; medial = 56%, SE = 3.8; final = 43% correct, SE = 2.7).

## General Discussion

The results of this study suggest that AV training can promote auditory perceptual learning of novel, vocoded speech more effectively than AO training. But the training procedure affects perceptual learning outcomes. In Experiment 1, PA training was carried out with disyllabic spoken nonsense words and nonsense pictures. Participants were assigned to learn the associations with either AV or AO speech stimuli within a fixed number of trials. AV training was significantly more effective than AO training, as measured by testing how well the paired associations could be identified with AO stimuli. Pre- and post-training forced-choice consonant identification was also administered AO with untrained sets of disyllabic spoken nonsense words. On this task also, AV-trained participants were more accurate than AO-trained participants. Perception of medial consonants was significantly affected by AV training. AV-trained participants gained 24% points accuracy for medial consonants, and AO-trained participants gained 17% points. In Experiment 2, a control experiment, participants were tested twice in the forced-choice consonant identification paradigm but without intervening training or feedback of any kind. Their re-test scores were significantly higher than their initial scores. The consonant identification scores were then compared across Experiments 1 and 2. The comparison showed that AO-trained participants in Experiment 1 were *no more* accurate on consonant identification than re-tested participants in Experiment 2. In contrast, AV-trained participants in Experiment 1 were *more* accurate than re-test participants in Experiment 2. Experiment 3 was carried out using PA training that alternated between AV and AO conditions on a list-by-list basis (mixed training). Training was to a 92% correct criterion, and two more lists were trained than in Experiment 1. Lists tested after AO training resulted in significantly higher AO PA scores than lists tested after AV training. Test scores on the paired associations were compared across Experiments 1 and 3. AV-trained participants in Experiment 1 were significantly more accurate (97% correct) than participants in Experiment 3 following AV training (88.9% correct). AO-trained participants in Experiment 1 performed similarly to participants in Experiment 3 following AO training (Experiment 1, 92% and Experiment 3, 94.0% correct).

### Reverse hierarchy theory for multisensory speech processing

The results of Experiment 1 suggest that multisensory stimuli can be used for improving unisensory perceptual learning. But the results of Experiment 3 suggest that multisensory stimuli can also impede unisensory perceptual learning. A theory of perceptual learning (Goldstone, 1998) is needed to explain these discrepant results. We have adopted the reverse hierarchy theory (RHT) of perceptual learning (Ahissar and Hochstein, 1997; Ahissar et al., 2008), because it attempts to explain perception and perceptual learning within the context of neural processing.

The *hierarchy* in *RHT* refers to the organization of visual and auditory sensory-perceptual pathways (Felleman and Van Essen, 1991; Kaas and Hackett, 2000). Although sensory-perceptual pathways are not strictly hierarchical, their organization is such that higher-levels show selectivity for increasingly complex stimuli combined with an increasing tolerance to stimulus transformation and increasing response to perceptual category differences (Hubel and Wiesel, 1962; Ungerleider and Haxby, 1994; Logothetis and Sheinberg, 1996; Zeki, 2005).

According to RHT, immediate perception relies on established high-level representations in the bottom-up sensory-perceptual pathway. When a new perceptual task needs to be carried out, naïve performance is initiated on the basis of immediate high-level perception. However, if the task cannot be readily performed with the existing mapping of low-level to high-level representations, and/or if there is incentive to increase the efficiency of task performance, then perceptual learning is needed. According to RHT, perceptual learning is the access to and remapping of lower-level input representations to higher-level representations. To carry out the remapping, perceptual learning involves “perception with scrutiny.” That is, a backward search must be initiated to access the representational level of the information needed to carry out the perceptual task. A new mapping can then be made. Mapping changes can occur in both convergence and divergence patterns (Jiang et al., 2007b; Kral and Eggermont, 2007; Ahissar et al., 2008). That is, dissimilar lower-level input representations can map to the same higher-level representations; and similar lower-level input representations can map to different higher-level representations.

### Speech processing pathways

**Figure 6:**
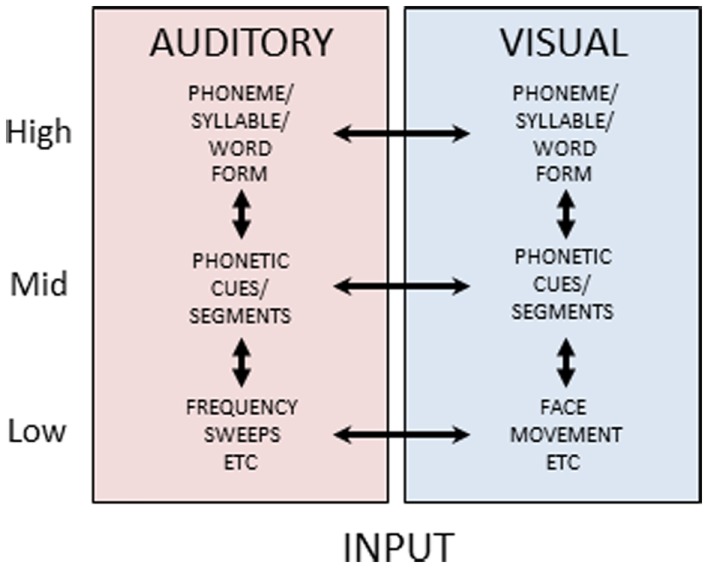
**Auditory and visual speech pathways**. Figure 6 schematically depicts cortical processing pathways and their interactions for auditory and visual speech. Separate uni-sensory processing pathways are thought to exist for mapping from low-level stimulus features to high-level form-based representations of speech stimuli. During perceptual processing, information is thought to predominantly flow from low-to-high, however feedback pathways are available along both pathways. Additionally, at each level double arrowed lines between the pathways indicate the potential for multisensory integrative processing.

Reverse hierarchy theory has not, to our knowledge, previously been extended to an explicit theory of multisensory constraints on unisensory perceptual learning, but the evidence on the diversity and extent of cortical and subcortical multisensory connections (Foxe and Schroeder, 2005; Ghazanfar and Schroeder, 2006; Driver and Noesselt, 2008; Kayser et al., 2012) suggests that higher-level representations in one sensory-perceptual system can be used to gain access to lower-level representations in another sensory-perceptual system. Figure 6 is a schematic view of auditory and visual speech processing pathways. It suggests that at each level of stimulus processing – basic features (e.g., spectrotemporal auditory features and spatiotemporal visual features not specific to speech), phonetic features (linguistically relevant sub-phonemic integrated basic features), phonemes (syllables or word forms, i.e., linguistically relevant categories) – there is the possibility of multisensory integrative processes and also unisensory representations. Various experimental results have been interpreted as evidence that visual speech information can converge as early as primary auditory cortex (e.g., Sams et al., 1991; Calvert et al., 1997; Giard and Peronnet, 1999; Möttönen et al., 2002; Raij et al., 2010), and anatomical animal studies have provided evidence of multisensory connectivity as low as primary visual and auditory areas (Ghazanfar et al., 2008; Falchier et al., 2012). Such results have been interpreted as support for early and obligatory multisensory integration (Rosenblum, 2008). Other findings point to multisensory integration at higher cortical levels, such as superior temporal sulcus, suggesting that extensive unisensory integration has occurred prior to integrative activity (Miller and D’Esposito, 2005; Hasson et al., 2007; Bernstein et al., 2008a; Nath and Beauchamp, 2011).

Figure 6 shows a parallel structure for unisensory auditory and visual speech processing. The parallel unisensory hierarchy for visual speech receives diverse support in the literature. For example, dissimilarity measures of visual speech stimuli significantly account for consonant perceptual dissimilarity (Jiang et al., 2007a; Files and Bernstein, in preparation). That is, physical optical measures can account for significant variance in visual perceptual identification and discrimination. Patterns of confusions for lipreading words are reliably accounted for by visual perception of spoken phonemes (Mattys et al., 2002). Visual perceptual confusions account for results on visual spoken word identifications better than auditory perceptual confusions (Auer, 2002). Visual speech mismatch negativity event-related potentials have been localized posterior to auditory temporal cortices (Ponton et al., 2009; Files and Bernstein, submitted), and visual speech processing has been localized with functional magnetic resonance imaging in posterior superior temporal cortex and adjacent middle temporal cortex, consistent with speech representation in the high-level vision pathway (Bernstein et al., 2011).

Thus, speech perception can be multisensory, visual-only, or auditory-only, and there is support for representations that correspond to these three possibilities. It also seems reasonable to conclude across the many results on speech perception involving auditory and visual stimuli that multisensory integration is available at every level of speech processing, consistent with a highly multisensory cerebral cortex (Ghazanfar and Schroeder, 2006). How could this diversity of integrative resources contribute to the discrepant results of Experiments 1 and 3?

### Explanation for divergent multisensory training outcomes

In order to explain our divergent results, we need to focus on the level at which auditory perceptual learning took place. Our results point to phonetic features, which are linguistically relevant sub-phonemic representations that typically are said to map to phoneme categories (for discussion of features, Jakobson et al., 1961; Chomsky and Halle, 1968) but could also map directly to syllable, morpheme, or word-level categories (Grossberg et al., 1997; Vitevitch and Luce, 1999; Norris et al., 2000). The results point to auditory perceptual learning of phonetic features, because learning generalizes to forced-choice consonant identification in new words, and learning is differentially affected by the position of the consonant. If consonants were learned as unanalyzed units, we would not expect that their position in the word would be a significant effect in our results. The medial consonant affords the most phonetic feature information, which is obtained from the vowel transitions into and out of the consonant (Stevens, 1998), and therefore phonetic feature learning should result in more gains when feature information is richer. In addition, the largest amount of auditory learning was for the medial consonant position following AV training: Auditory perceptual learning was more sensitive to phonetic details in the auditory stimuli when the training was AV.

To be clear, phonetic features are integrated representations based on basic sound features. That phonetic features are complex combinations of information about the acoustic attributes of speech has been extensively researched (Stevens, 1998). For example, the place of articulation (e.g., involved in the distinction /b/ versus /d/) is instantiated in the acoustic signal partly by the center frequency and transitions of the speech formants (resonance of the vocal tract). The feature known as voicing (e.g., involved in the distinction /b/ versus /p/) is instantiated partly by the temporal offset difference between consonant initiation in the supralaryngeal vocal tract and the onset of glottal pulsing (Lisker et al., 1977). Relatively little research has been carried out on the neural bases of phonetic feature processing, with most speech perception research focused on levels either lower than or higher than phonetic features (Binder et al., 2000; Scott, 2005; Hickok and Poeppel, 2007; Liebenthal et al., 2010), however, Obleser and Eisner (2009) have identified a site of phonetic feature processing anterior to the primary auditory cortical areas in superior temporal gyrus. This gives support to the possibility of focused phonetic feature learning.

When speech is degraded or transformed, perceptual confusions among phonemes can be described in terms of loss of phonetic feature distinctions (Miller and Nicely, 1955; Wang and Bilger, 1973). The problem for auditory perceptual learning of vocoded speech is to remap available basic auditory features (such as frequency and temporal features) in the novel transformation to phonetic features that support the perception of syllables, morphemes, and/or words.

**Figure 7:**
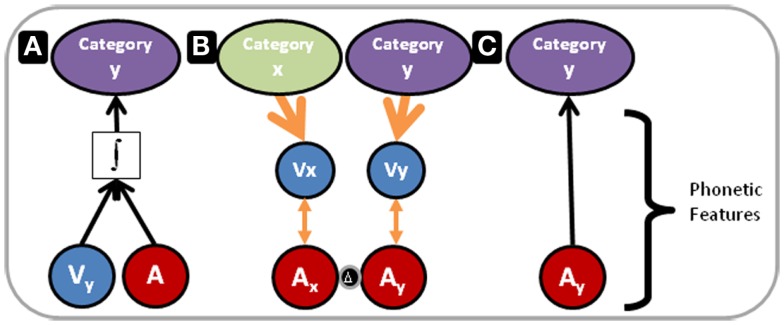
**Perceptual learning versus integration model**. The blue and red circles in the lower part of Figure 7 represent visual and auditory phonetic speech features, respectively. These correspond to the mid level of processing in Figure 6. The categories at the top of the figure correspond to representations at the high-level of processing in Figure 6. **(A)** Depicts processing under conditions in which acoustic phonetic features alone are not sufficient to specify the phoneme category. The integrated audiovisual phonetic features do provide adequate information. Perceptual processing flows bottom-up, and remapping along the auditory pathway has not occurred. In contrast, **(B)** Depicts a reverse flow of information. As in **(A)**, Combined audiovisual information is sufficient to specify phoneme categories (not shown). However, here a reverse search is initiated. Higher-level visual speech categories, *x* and *y*, feed back to visual phonetic features, *Vx* and *Vy*, that use natural audiovisual correlations (orange double arrowed lines) to guide the search for relevant distinctions in acoustic-phonetic feature representations. The two red circles separated by a delta are labeled *Ax* and *Ay* because the acoustic phonetic features are now distinct. **(C)** Depicts auditory-only processing, following the perceptual learning depicted in **(B)**. The acoustic phonetic features alone are now sufficient to specify the phoneme category.

Figure 7 illustrates our proposed model for the outcomes of Experiments 1 and 3 within the context of multisensory and unisensory processing resources and the RHT of perceptual learning. In Figure 7, the blue and red circles represent visual and auditory phonetic speech features, respectively. For purposes here and in Figure 7, the category that phonetic features target is not important to define, because the results of the three experiments point to auditory perceptual learning at the phonetic feature level targeting phonemes, and as pointed out above features could target phonemes, syllables, morphemes, or words.

In Figure 7A, vocoding has removed or distorted the basic auditory information that is typically mapped to phonetic features of natural speech. The phonetic feature level is inadequate to specify the phoneme category (phoneme categories for purposes here). But the visual speech information provides the needed phonetic information (Summerfield, 1987), the information is integrated, and the perceptual task is carried out at an immediate high-level of perception, as predicted by RHT. However, with early integration the perceptual task can be accomplished without scrutiny of auditory lower-level representations, and if the visual stimulus is unavailable performance drops. This is our explanation for the finding in Experiment 3, in which performance following AV training was lower than following AO training.

Several factors in Experiment 3 could have reduced the likelihood that participants focused on the auditory information when the training was AV. RHT predicts that when semantic processing is required, low-level access is precluded (Ahissar et al., 2008; Nahum et al., 2008). In Experiment 3, participants were trained to criterion, and they were free to train on as many lists as possible during a training session. Trying to learn more than one list in a day could have directed attention to semantic relationships. Training to criterion on more than one list could have encouraged less attention to the auditory input, because it might have led participants to put a premium on the rate at which the paired associations were learned rather than on the accuracy of the AO tests. Also, given that perception of AV speech stimuli is frequently faster and more reliable (Sumby and Pollack, 1954; Van Wassenhove et al., 2005; Ross et al., 2007), we surmise that in Experiment 3 the perceived effort to learn the paired associations was lower under AV versus AO conditions. This perceived reduced effort might have also favored relying on high-level representations that were fed by AV integration. While it is true that semantic category training can result in retuning representations (Jiang et al., 2007b) and change in sensitivity to category boundaries (Goldstone, 1994), such training typically involves less diverse stimuli than the ones in the present study.

Figure 7B has two columns. Each has a downward arrow from a higher-level of visual speech category representation to a level that is correlated with auditory representations. Remapping from basic sound to phonetic features has taken place due to top-down guidance within the visual system. The red circles are labeled *Ax* and *Ay*, because phonetic features are now distinct. We think that the auditory distinctions that were learned in our study *must* be readily available at the level of basic features (not indicated in Figure 7), because learning was relatively fast and low-level auditory retuning is likely not affected over such a brief period (Kral and Eggermont, 2007). Likewise, the rapid learning argues against learning based on new connections via dendritic growth and arborization.

We hypothesize that this remapping process makes use of natural correlations between auditory and visual speech stimuli, indicated in Figure 7B with the double pointed arrows. These natural AV correlations provides a link whereby visual information can help guide attention to the relevant distinctions in the auditory representations. Research on the predictability of acoustic signals from optical signals and *vice versa* has shown that there are high-levels of correlation between acoustic and optical speech signals (Yehia et al., 1998; Jiang et al., 2002; Jiang and Bernstein, 2011). Perceptual evidence shows that quantified correlation of the physical acoustic and optical speech signals can account for AV speech responses with matched and mismatched (McGurk type) stimuli (Jiang and Bernstein, 2011). Visual speech stimuli have been suggested to modify auditory speech processing through modulatory effects on neuronal excitability (Schroeder et al., 2008). Speech-in-noise experiments suggest that perceivers adjust their perception and neural networks change in relationship to the relative reliability of auditory or visual information (Ross et al., 2007; Nath and Beauchamp, 2011), or the temporal alignment of the stimuli (Miller and D’Esposito, 2005). We are suggesting that top-down processing from visual speech representations can guide access to distinctive auditory features that can be remapped to phonetic features for novel speech transformations. Top-down guidance via orthographic representations has been suggested as another basis for auditory perceptual learning of vocoded speech (Davis et al., 2005).These two types of top-down guidance might result in different learning. Specifically, the multisensory speech correlations might provide more fine-grained guidance for phonetic learning than orthography.

In Figure 7C, following the successful remapping, when AO stimuli are presented, the auditory mapping to the category is sufficient to carry out the task. Figure 7C corresponds to the result in Experiment 1 that AV PA training was more effective than AO training or merely re-testing in Experiment 2.

### Some implications for training

Results reported here could be important clinically, for example, to crafting strategies for patients newly fitted with a cochlear implant (Zeng et al., 2004). The goal of such training is to assist the cochlear implant user in gaining access to the information in the degraded or impoverished signal delivered by the auditory prosthesis. Such patients can benefit from auditory training, but the benefits are typically not large (Fu et al., 2005; Stacey et al., 2010). A focus in training studies has been on which linguistic units such as phonological features, syllables, words, or sentences might best promote auditory perceptual learning (Fu et al., 2005; Stacey et al., 2010). However, the goals of training might be better served by focusing on the flow of information processing, specifically, the possibility that reverse hierarchy processing is needed to gain access to the available information (Kral and Eggermont, 2007; Auer and Bernstein, 2012). Focus is needed on the possibility that top-down guidance must be crafted that allows access to the level of representation where additional cues are available to be remapped. The current results support this view. But knowledge is also needed to predict when AV integration can impede auditory perceptual learning.

The results here are particularly relevant to training young cochlear implanted children who have not yet learned to read. In contrast to literate normal-hearing adults who can use orthographic representations or clear speech to guide perceptual learning (Davis et al., 2005; Hervais-Adelman et al., 2011), children’s guides are often limited to multisensory information delivered via lipreading, visual signed language or fingerspelling, and/or vibrotactile speech displays (Bernstein et al., 1991; Auer et al., 1998).

A concerted effort was made in the twentieth century to design and test vibrotactile speech perception prostheses to supplement lipreading by deaf individuals including children. While the intent of the research was to learn how to convey speech through mechanical vibration signals, combined visual-vibrotactile training was shown to be associated with improved visual-only speech perception (Boothroyd and Hnath-Chisolm, 1988; Eberhardt et al., 1990; Bernstein et al., 1991; Kishon-Rabin et al., 1996). These improvements in lipreading sometimes exceeded the vibrotactile learning. This type of result suggests that when a novel speech signal is combined with a more familiar one, attention might be directed toward discerning additional information from the more familiar signal rather than the target novel signal. Indeed, in a companion study (in preparation) to this one on prelingually deaf adults who obtained cochlear implants as adults, we found that AV training resulted in faster PA learning but poorer auditory-only test scores, consistent with attention to and reliance on the more familiar visual stimuli. Indeed, there is evidence that visual perceptual abilities and multisensory integration are affected by cochlear implant usage in adults (Rouger et al., 2007). Understanding is needed for how to devise training that uses multisensory stimuli to guide unisensory perceptual learning, rather than only effecting immediate high-level perception with concomitant failure to achieve discernment of available low-level distinctions.

## Summary and Conclusion

In summary, the results reported here do not fall under the rubrics of faster or more accurate AV versus AO speech perception, effects that have been well-documented (e.g., Sumby and Pollack, 1954; Bernstein et al., 2004; Van Wassenhove et al., 2005; Ross et al., 2007). They concern AV versus AO training effects on auditory-only perceptual learning. The information in a visual speech stimulus, presented in synchrony with a correlated but degraded auditory stimulus, can be effective in promoting auditory speech perceptual learning of the degraded stimuli. The visual information can promote more learning than the auditory stimuli alone, because of the correlations between auditory and visual features or cues, and because top-down visual processes can guide access to available but unused auditory cues. However, the multisensory speech stimuli typically are more informative and easier to perceive, and multisensory perception can rely on integrated representations, thereby possibly impeding unisensory perceptual learning. Research is needed on what perceptual learning procedures are required so that multisensory stimuli can be used reliably to enhance unisensory perceptual learning.

## Conflict of Interest Statement

The authors declare that the research was conducted in the absence of any commercial or financial relationships that could be construed as a potential conflict of interest.
